# Addressing Aortopathy Associated With Congenital Valve Anomalies: Technical Aspects of a Reoperative Bentall-Konno Procedure

**DOI:** 10.1177/21501351261418293

**Published:** 2026-03-05

**Authors:** Ryan E. Accord, Martijn D. Gilbers, Elham Bidar

**Affiliations:** 1 Department of Cardiothoracic Surgery, Center for Congenital Heart Disease, 10173 University Medical Center Groningen, University of Groningen, Groningen, the Netherlands; 2 Department of Cardio-Thoracic Surgery, 199236 Cardiovascular Research Institute Maastricht, Maastricht University Medical Center+, Maastricht, the Netherlands

**Keywords:** aortic annular enlargement, aortic root replacement, BAV aortopathy, congenital valve anomaly

## Abstract

Children and adolescents with congenital aortic stenosis often need multiple reoperations due to early intervention and somatic growth. When valve replacement is considered, selecting an optimally sized prosthesis is essential. The Konno aortoventriculoplasty enables annular enlargement to accommodate larger valves. Patients with unicuspid or bicuspid valves are increasingly recognized as having intrinsic and extrinsic risk factors for aortopathy, sometimes progressing to aneurysmal degeneration requiring concomitant root replacement. We describe a surgical approach combining the Konno procedure with a Bentall operation for selected patients.

## Background

Young patients with congenital aortic valve stenosis who require early valve replacement face a lifelong risk of reoperation.^1^ Two issues predominate:

One major challenge is the small size of the native aortic annulus. Early intervention often leaves the annulus too small for an adult-sized prosthesis, leading to patient-prosthesis-mismatch (PPM) as the patient grows. Beyond supra-annular placement of oversized prostheses, annular enlargement techniques are frequently employed to implant larger valves during both initial and redo surgeries. Posterior techniques such as the procedures described by Manouguian and Nicks can achieve an increase of one to two valve sizes.^2,3^ In contrast, the Konno procedure involves anterior enlargement via a right ventricular outflow tract (RVOT) ventriculotomy, allowing placement of a prosthesis up to three sizes larger.^4–6^

Another concern is aortic root pathology, particularly in patients with bicuspid or unicuspid aortic valves, in whom intrinsic aortic wall abnormalities are increasingly recognized as the underlying cause.^7^ Although significant root dilatation is uncommon during childhood surgery, it should be carefully evaluated at reoperation, especially in the presence of additional risk factors.^8^ Moreover, the supra-annular placement of oversized prostheses may bring the device close to the coronary ostia, complicating redo valve replacement. In such cases, aortic root replacement may be required, with coronary button mobilization and reimplantation providing a safe reconstructive option.

We describe, step-by-step, the combination of a Konno procedure with a Bentall operation in a 31-year-old patient with PPM and aortopathy, with two additional illustrative cases included in the Supplement.

## Case Example

A 31-year-old female (BSA 1.95 m^2^) with congenital aortic valve stenosis presented with dyspnea and exercise intolerance. She was previously treated with balloon valvuloplasty in infancy, followed by aortic valve replacement at the age of 22 with a 19-mm bioprosthesis (Carpentier-Edwards Perimount Magna Ease). The valve was implanted in a supra-annular position, and the sinus of Valsalva was enlarged to prevent interference by the prosthesis with the left coronary artery. In hindsight, even with the attempt to implant an oversized valve, the occurrence of PPM could have been foreseen. At presentation, transthoracic echocardiography showed severe aortic stenosis with an aortic valve area of 0.6 cm^2^ and a peak velocity of 4.8 m/s. In addition, computed tomography revealed ascending aortic dilatation, with a maximum diameter of 45 mm.

After discussing surgical options, the patient opted for mechanical valve replacement rather than a Ross-Konno procedure. At reoperation, the aortic tissue was extremely friable, requiring root replacement in addition to annular enlargement. A modified Bentall with Konno enlargement was performed using a 25-mm CarboSeal Valsalva conduit. Recovery was uneventful, and the patient was discharged 7 days postoperatively in good condition. At 4-year follow-up, the patient remains in good clinical condition and free of complaints.

## Surgical Technique

**Figure 1.**
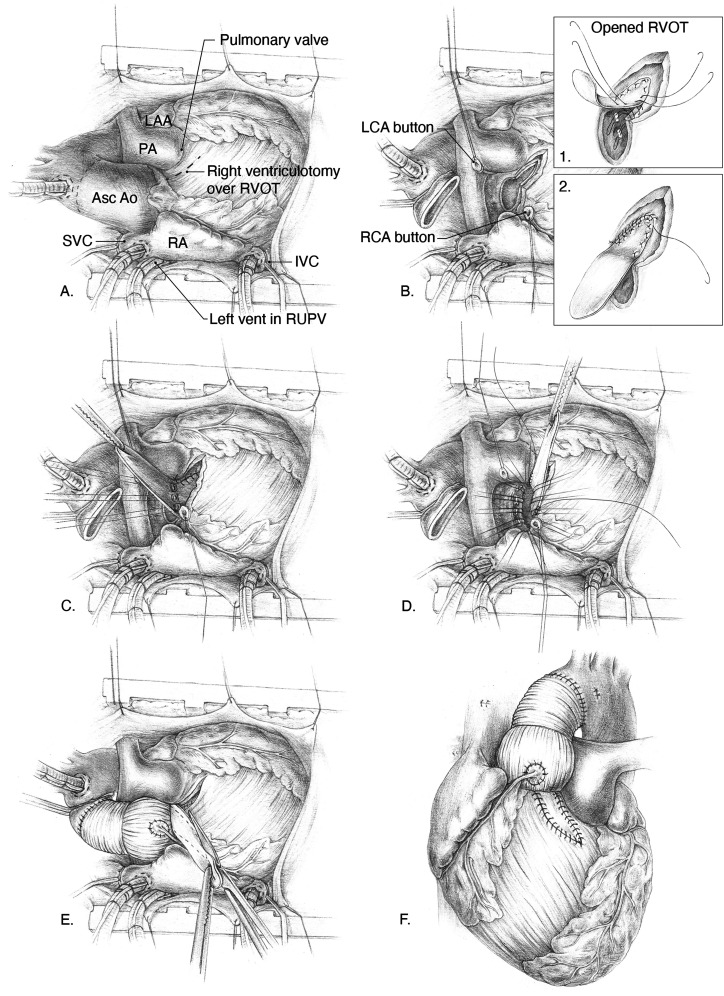
(A-F) Surgical steps of the Bentall-Konno procedure: (B) After excision of the previous prosthesis and aneurysmal aorta, a Konno incision is made in the outlet portion of the interventricular septum (IVS). Patch enlargement is performed with bovine pericardium using pledgeted sutures, followed by a continuous Prolene suture (Windows 1 and 2). (C) Valve sutures are placed through the patch. (D) Additional sutures are placed in an everting manner in the remaining native annulus. (E) Root replacement is completed, followed by reconstruction of the anterior right ventricular outflow tract (RVOT) using the remaining patch. Abbreviations: Asc Ao, ascending aorta; IVC, inferior vena cava; LAA, left atrial appendage; LCA, left coronary artery; PA, pulmonary artery; RA, right atrium; RCA, right coronary artery; RUPV, right upper pulmonary vein; RVOT, right ventricular outflow tract; SVC, superior vena cava. Practical Pearls & Pitfalls:

All key surgical steps of the Bentall-Konno procedure are illustrated in Figure 1. After aortic arterial and bicaval venous cannulation, cardiopulmonary bypass is initiated. Bicaval cannulation enables total bypass and a bloodless operative field when opening the RVOT. The base of the aortic root is dissected to the level of the annular plane. After aortic cross-clamping and cardioplegic arrest, all friable aortic root tissue and the bioprosthesis are excised. The RVOT is incised anteriorly, avoiding visible conal coronary branches and remaining approximately 8 to 10 mm from the pulmonary valve. After confirming the position of the pulmonary valve, the incision is extended rightward toward the interventricular septum, remaining left of the coronary button. The aortic annulus and infundibular septum are then incised (Konno incision) under direct vision along with the expected left coronary cusp-right coronary cusp (LCC-RCC) commissure, away from the membranous septum beneath the RCC-noncoronary cusp (NCC) interleaflet triangle. The incision is also kept left of the conus papillary muscle of the tricuspid valve (Muscle of Lancisi), which lies adjacent to the bundle of His, all of which is intended to avoid injury to the conduction system (Figure 1B). The required augmentation for adequate prosthesis and patch width is determined. A single bovine pericardial patch is used to reconstruct the ventricular septal defect. To provide a practical reference for tailoring patch width at the annular level, upsizing from a 19-mm prosthesis by three sizes increases the diameter by approximately 6 mm. This corresponds to an approximately 19 mm increase in left ventricular outflow tract circumference (6 × π), with an additional ∼3 mm margin on both sides for secure patch suturing. A teardrop-shaped patch, with an approximately 25 mm base, is secured using a series of pledgeted mattress sutures (Ticron 2-0) for reinforcement, followed by a running suture (Prolene 4-0), initiated at the end of the Konno incision, working toward the annulus (see detail windows). Pledgeted annular valve sutures are then placed with the pledgets on the RVOT side of the patch (Figure 1C). The remaining pledgeted annular sutures in the NCC and LCC cusp regions are placed according to preference, using either the everting or non-everting technique (Figure 1D). Standard Bentall steps are performed, including prosthesis sizing, conduit placement, and coronary button reimplantation. The remainder of the bovine patch is trimmed (Figure 1E) to reconstruct the anterior part of the RVOT using a single-layer running suture (Prolene 4-0) (Figure 1F). This part of the procedure may be performed on a beating heart if preferred. Particular attention should be directed to the aortoventricular junction (*), which may require additional reinforcement prior to aortic declamping. The default in our practice is to begin the first 1 to 2 cm of the RVOT patch reconstruction (**) before releasing the aortic cross-clamp, allowing early myocardial reperfusion. Alternatively, one may choose to complete the entire RVOT reconstruction first, followed by root replacement.

• During the RVOT incision and patch placement, avoid visible conal coronary branches. • Stay clear of the membranous septum to prevent conduction system injury. •Address the critical area at the aorto-ventricular junction (*) before exposure becomes limited: begin the first 1 to 2 cm of RVOT patch reconstruction (**) before declamping the aorta.

## Comments

We describe an effective, reproducible technique for Konno aortoventriculoplasty combined with a Bentall procedure to address small annular size and aortopathy in congenital valve anomalies. The anterior aortoventriculoplasty was first described independently by Konno and Rastan. While congenital surgeons may be familiar with the Konno procedure, including its conduction-system-related risks, the attendant risk of pacemaker implantation, and strategies to mitigate these risks, the need for concomitant root replacement in this population has only been increasingly recognized over the past two decades. The root phenotype of bicuspid aortic valve (BAV)-associated aortopathy is typically observed in younger patients and is associated with higher rates of complications. The interrelation between coarctation, BAV, and aortopathy underscores that a dilated root cannot be ignored, particularly as some young patients exhibit persistent hypertension as an added risk factor. Recent guidelines for adults, as well as those for children and adolescents, state that BAV-associated ascending aortic dilatation should be addressed at a diameter of 45 mm if aortic valve repair or replacement is planned.^7,8^ However, in younger or small-BSA patients, absolute aortic diameters often underestimate aortopathy severity, making BSA-indexed Z-score considerations (typically >+3 to +4) essential for guiding the timing of root replacement and optimizing prosthesis and graft sizing. Beyond BAV aortopathy, full root replacement is also suited for reconstructing the aorto-ventricular junction in endocarditis complicated by fistula or abscess, or in redo root replacement after a previous Ross-Konno (see Supplement cases). In the latter, a simple Konno-type patch enlargement suffices, whereas infective cases necessitate radical debridement and complex patch reconstruction. Furthermore, root replacement may be required in redo cases in which a coronary ostium lies close to a previously supra-annular, oversized prosthesis.

In conclusion, in patients with congenital valve anomalies and a small aortic annulus in whom clinically relevant aortopathy is present, the Bentall-Konno procedure offers a tailored and reproducible solution. It is equally applicable in complex redo settings requiring reconstruction of the aortic root and aortoventricular junction, such as infective endocarditis or late (pseudo)aneurysmal complications. When performed with meticulous attention to conduction-system preservation, it represents an essential addition to the contemporary armamentarium of pediatric and adult cardiothoracic surgeons.

## Supplemental Material

sj-docx-1-pch-10.1177_21501351261418293 - Supplemental material for Addressing Aortopathy Associated With Congenital Valve Anomalies: Technical Aspects of a Reoperative Bentall-Konno Procedure Supplemental material, sj-docx-1-pch-10.1177_21501351261418293 for Addressing Aortopathy Associated With Congenital Valve Anomalies: Technical Aspects of a Reoperative Bentall-Konno Procedure by Ryan E. Accord, Martijn D. Gilbers and Elham Bidar in World Journal for Pediatric and Congenital Heart Surgery

sj-docx-2-pch-10.1177_21501351261418293 - Supplemental material for Addressing Aortopathy Associated With Congenital Valve Anomalies: Technical Aspects of a Reoperative Bentall-Konno Procedure Supplemental material, sj-docx-2-pch-10.1177_21501351261418293 for Addressing Aortopathy Associated With Congenital Valve Anomalies: Technical Aspects of a Reoperative Bentall-Konno Procedure by Ryan E. Accord, Martijn D. Gilbers and Elham Bidar in World Journal for Pediatric and Congenital Heart Surgery
