# Supplementary material for: Addressing Aortopathy Associated With Congenital Valve Anomalies: Technical Aspects of a Reoperative Bentall-Konno Procedure
Source: World J Pediatr Congenit Heart Surg. 2026 Mar 5;17(5):672–5. doi: 10.1177/21501351261418293 (PMC13443385; doi:10.1177/21501351261418293)
Supplement: Supplementary file 1 [file sj-docx-1-pch-10.1177_21501351261418293.docx]

**Supplement 1:** An additional case of redo Bentall-Konno procedure in the setting of infective endocarditis.

A 25-year-old asymptomatic male patient (BSA 2.07 m^2^) was referred following the incidental finding of systolic and diastolic turbulence in the right ventricular outflow tract (RVOT) on transthoracic echocardiography. His medical history was notable for congenital valvular and subvalvular aortic stenosis, for which he had undergone a commissurotomy and subvalvular membrane resection at the age of 5. He subsequently received a 25-mm CarboMedics mechanical aortic valve after undergoing a Konno procedure to enlarge the aortic annulus.

Further evaluation of the RVOT turbulence revealed an aortic root aneurysm with a significant shunt into the RVOT. Given the severity of the findings, the patient was scheduled for urgent surgical intervention.

During the reoperation, a pseudoaneurysm of the aortic root and a large fistula to the RVOT were identified (Image: 3D reconstruction based on CT angiography). Additionally, there were signs suggestive of a low-grade infection. Extensive debridement was carried out, and swabs for microbial cultures were obtained. The previously placed Konno patch was replaced, and a 25-mm CarboSeal Valsalva mechanical Bentall composite conduit was implanted as described in the manuscript. Although pulmonary root/valve replacement with a homograft was anticipated, RVOT integrity could be restored with the new Konno patch, allowing preservation of the native pulmonary valve.Despite negative results from both blood and tissue cultures, ceftriaxone was continued postoperatively due to a strong clinical suspicion of infection. The patient’s postoperative course was uneventful, and he was discharged after a 14-day hospitalization. Follow-up CT at 6 months demonstrated an intact valved conduit without complications. At 30 months, the patient remains in good clinical condition without any symptoms.

Pre-operative 3D CT reconstruction.

Post-operative 3D CT reconstruction.
