# Supplementary material for: Addressing Aortopathy Associated With Congenital Valve Anomalies: Technical Aspects of a Reoperative Bentall-Konno Procedure
Source: World J Pediatr Congenit Heart Surg. 2026 Mar 5;17(5):672–5. doi: 10.1177/21501351261418293 (PMC13443385; doi:10.1177/21501351261418293)
Supplement: Supplementary file 2 [file sj-docx-2-pch-10.1177_21501351261418293.docx]

**Supplement 2:** An additional case of redo Bentall-Konno procedure in the setting of autograft dilation and aortic regurgitation 17 years after unsupported Ross procedure.

A 60-year-old female patient was referred to our center with progressive combined aortic regurgitation and stenosis, along with moderate pulmonary valve stenosis. She had a history of congenital valvulopathy, previously treated with an unsupported Ross procedure combined with resection of a subvalvular membrane 17 years earlier at another center. At presentation, the neoaortic root (autograft) was dilated (maximum diameter 52 mm), resulting in central aortic regurgitation and progressive left ventricular dilatation. Echocardiography showed an aortic valve peak gradient of 60 mmHg and a pulmonary valve peak gradient of 42 mmHg.
The planned procedure was aimed at preservation of the neo-aortic valve (valve repair or valve-sparing root replacement) or a Bentall procedure, with or without concomitant pulmonary valve replacement.

On resternotomy, extensive adhesions were encountered, especially between the autograft and the pulmonary homograft, necessitating replacement of the pulmonary root.
After cardioplegic arrest, the dilated ascending aorta was opened, confirming poor aortic wall quality. The aortic cusps were thickened, with prolapse of the left coronary cusp. A durable repair was considered unlikely. Consistent with the preoperative discussion with the patient, a mechanical Bentall procedure was performed. A restrictive subvalvular membrane was resected, which likely contributed to cusp thickening. Given the narrow LVOT, a Konno enlargement of the aortic annulus and LVOT was carried out, and reconstructed with a patch. This allowed implantation of a 25-mm mechanical CarboSeal Valsalva prosthesis. The pulmonary root was replaced with a 24-mm pulmonary homograft. As the homograft already provided sufficient RVOT continuity, additional patch reconstruction of the anterior part of the RVOT was unnecessary.

At 9-year follow-up, the patient is in reasonable clinical condition, with mild heart failure attributed to chronic atrial fibrillation. In addition to rhythm management with His bundle ablation and pacemaker implantation, no additional cardiovascular interventions were required.
